# The cephalic vein is readily accessible for vascular access in pediatric patients less than 12 weeks presenting to an emergency room

**DOI:** 10.3389/fvets.2025.1495039

**Published:** 2025-03-20

**Authors:** Alexandra L. Zilberfarb, Adesola Odunayo, Prathima Garudadri, Ashley Allen-Durrance, Chika Okafor

**Affiliations:** ^1^Department of Small Animal Clinical Sciences, University of Florida, Gainesville, FL, United States; ^2^Department of Biomedical and Diagnostic Sciences, University of Tennessee, Knoxville, Knoxville, TN, United States

**Keywords:** intravenous catheter, puppies, kittens, cephalic vein, jugular vein

## Abstract

Pediatric veterinary patients often require prompt medical intervention in emergency hospitals, commonly involving intravenous medications or fluids. This study aimed to characterize the most utilized venous access sites in pediatric puppies and kittens under 12 weeks of age in an emergency room setting. Ninety-four canine and 33 feline patients under 12 weeks old, with an intravenous catheter placed in the emergency room at the University of Florida, between January 1, 2021, and November 30, 2023 were included in this study. A retrospective evaluation of medical records was conducted to determine the most common vein utilized for venous access in pediatric patients. The median body weight was 4.19 kg (Q1, Q3: 2.50, 7.20) for puppies and 0.92 kgs (Q1, Q3: 0.43, 1.14) for kittens. The cephalic vein was the most common site in both species, utilized in 90.4% of puppies (85/94) and 78.8% of kittens (26/33). Puppies with lower body weights were more likely to have a catheter placed in the jugular vein; however, no significant correlation was found between the kittens’ body weights and catheter site. The most used catheter size was 22G in both species. Ultimately, the cephalic vein appears to be consistently accessible for venous access in puppies and kittens under 12 weeks old, across a wide range of body weights. Catheters in the jugular vein may be preferred for puppies and kittens under 1.2 kg and 0.3 kg, respectively. Utilizing the cephalic vein for venous access may facilitate rapid and less technically challenging intravenous catheter placement in patients under 12 weeks old.

## Introduction

Pediatric veterinary patients are commonly presented to veterinary hospitals for evaluation of serious and sometimes life-threatening disorders including hypoglycemia, hypotension, severe dehydration, sepsis and/or anemia (1, 2). Obtaining prompt venous access is an important intervention in critically ill pediatric patients to aid in fluid resuscitation or administration of life-saving medications like fluids or dextrose. There is a lack of consensus in the definition of neonates/pediatrics in the veterinary literature with the neonatal period being defined as birth through 4 weeks, birth through 84 days, or birth through weaning (2, 3). The American Animal Hospital Association (AAHA) 2021 Canine Life Stage guidelines define the neonatal period as the time of birth to weaning (3–4 weeks of age) and the pediatric period is defined as the time of birth to sexual maturity (4–6).

In children, peripheral intravenous access is well established to be challenging with only about 41–73% of children successfully cannulated on the first attempt (7–10). Potential reasons for difficult venous access (DiVA) in children include small veins, undeveloped or anomalous surface vasculature, absence of visible or palpable veins and poor patient compliance (11–13). Optimal locations for establishing venous access have been described in children in an effort to reduce the number of venipuncture attempts made (14, 15). One advanced technique that has been developed to enhance the placement of peripheral intravenous catheters in pediatric DiVA patients is the ultrasound-guided approach. This method tends to have a better success rate than the standard visual or palpation technique (16). In veterinary practice, improvements in ultrasound guidance as well as progressive intraosseous access techniques have helped to improve and allow for more advanced venous access in challenging cases (17).

Establishing intravenous access is anecdotally thought to be challenging in pediatric veterinary patients as well, although no specific research has evaluated the success of venous access, as well as vessels that are easier to catheterize in pediatric puppies and kittens. Understanding the most accessible vessels may lead to less venipuncture attempts in puppies and kittens and swifter resuscitation efforts. The objective of this study is to describe the most common veins utilized in pediatric puppies and kittens less than 12 weeks of age in an emergency room. We hypothesize that the jugular vein will be the most common site of venous access in pediatric patients less than 12 weeks of age.

## Materials and methods

A review of the medical records of puppies and kittens with intravenous catheters placed in the Emergency Room (ER) at the University of Florida between January 2021 through November 2023 was carried out. Data was obtained from an automated query of the University of Florida’s Small Animal Hospital electronic health record^1^ and electronic treatment sheets.^2^ Inclusion criteria included pediatric puppies and kittens less than 12 weeks of age with an intravenous catheter placed in the ER. Intravenous catheters were defined as any short (≤1.5 inches) small bore (≤18G) catheter placed in a vein. Patients where the medical record was unclear as to where the catheter was placed were excluded from the study.

Information obtained for review from the medical records included patient signalment at the time they were seen, size and location of catheter placed, reason for intravenous catheter placement, when the catheter was removed and reason it was replaced (if applicable). The number of attempts made before the catheter was successfully placed was also recorded when available.

A commercially available statistical program^3^ was used to evaluate the data. The Kolmogorov–Smirnov test was used to access the normality of the continuous data (weight and age) of the patients and indicated that the data were non-parametric. Descriptive statistics of the measured variables were summarized as frequencies, proportions, means, standard deviations and medians with [lower (Q1), upper (Q3) quartiles]. For analytical statistics, a Kruskal Wallis test was performed to evaluate the difference in median body weight of patients among the groups evaluated (catheter size utilized and site of catheterization). A *p* value of <0.05 was considered significant.

## Results

Ninety-eight puppies and 33 kittens met the inclusion criteria for the study. Four puppies were excluded due to incomplete medical records which did not specify IV catheter size and/or location. No cats were excluded from the study. The number of attempts made before IV catheter placement was not documented for any patient.

### Canine data

**Figure 1:**
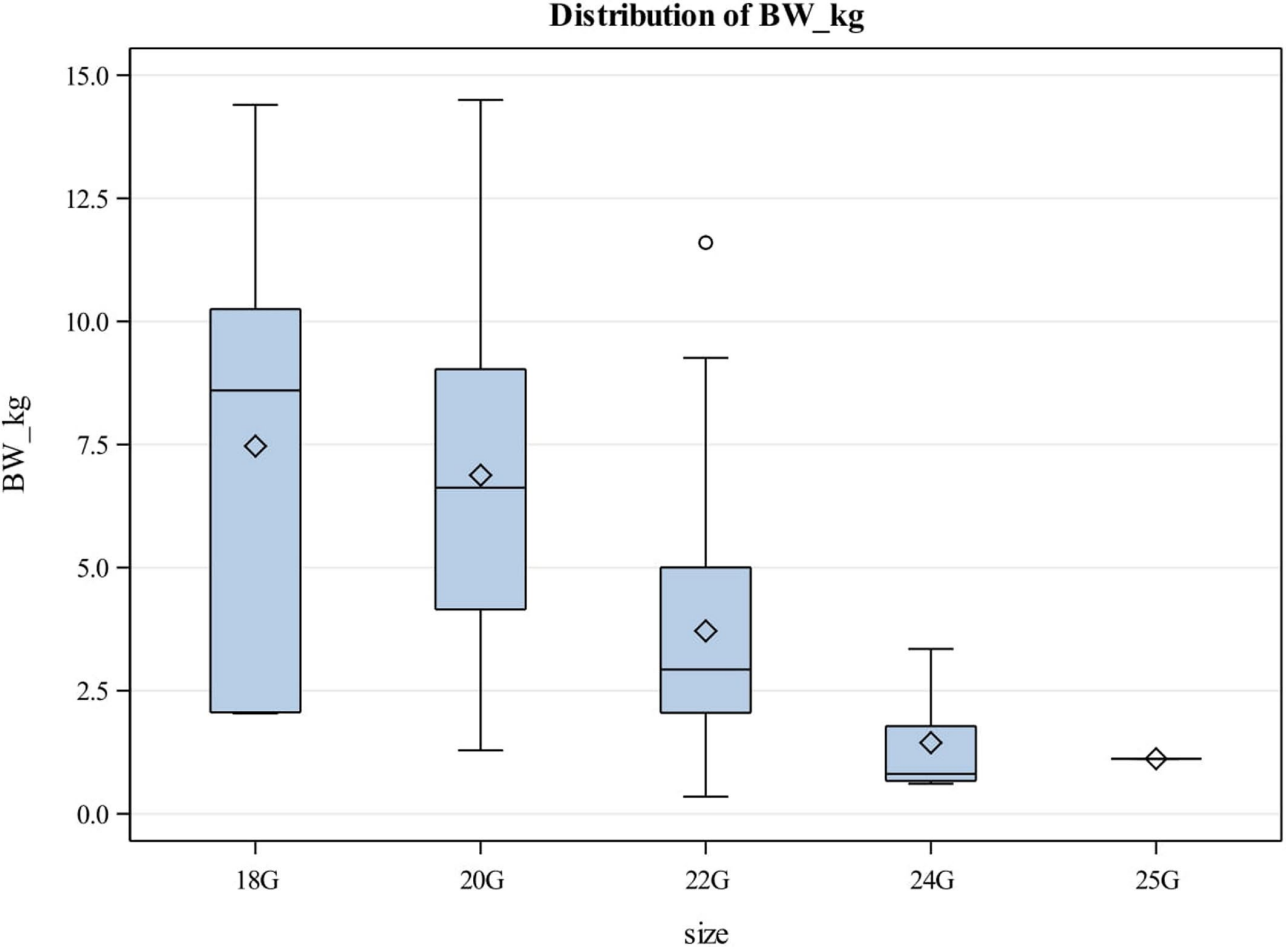
Box and Whisker plot showing relationship between body weight and catheter size utilized in puppies. The center line represents the median value (Q2 or the 50th percentile) and the diamond represents the mean. The box indicates the lower (QI or the 25th percentile) and upper (Q3 or the 75th percentile) quartiles while the whiskers indicate the 5th and 95th percentiles. Values beyond the upper and lower boundaries are outliers.

Forty-five female and 49 male puppies were enrolled in the study. The most common breeds represented were mixed breed dogs (15, 15.96%), followed by Pitbulls (6, 6.38%), German Shepherd dogs, Labrador Retrievers and Shih Tzus (4 each, 4.26%). Puppies had a median age of 10 weeks (Q1, Q3: 8, 12) and a median body weight of 4.19 kg (Q1, Q3: 2.50, 7.20). The most common reason for intravenous catheterization was for hospitalization (84, 89.4%), blood transfusions (6, 6.4%), sedation (3, 3.2%) and euthanasia (1, 1.1%). The most common catheter size utilized were 22G catheters (Table 1). Most catheters (85, 90.4%) were placed in a cephalic vein (Table 2). There was a significant association between the body weight of puppies and the catheter size used (*p* < 0.05) (Table 1; Figure 1). There was also a significant association between the body weight of puppies and the location of catheter placement (*p* < 0.05) (Table 2). Puppies with lower body weights were more likely to have a catheter placed in the jugular vein but there was no association between body weight and the placement of cephalic or saphenous catheters (Table 2).

**Table 1:** Association between animal weights and distribution of catheter sizes utilized in puppies and kittens presented to a veterinary emergency room.

Catheter sizes	18G	20G	22G	24G	25G
Puppies					
Mean weight (SD) (kg)	7.47 (5.38)	6.88 (3.31)	3.71 (2.46)	1.44 (1.17)	1.12 (0)
Median weight (Q1, Q3) (kg)	8.60 * (2.06, 10.25)	6.63 † ‡ (4.15, 9.03)	2.93 (2.05, 5.01) *‡	0.81 * † (0.67,1.78)	1.12 (1.12,1.12)
Number (%)	5 (5.3%)	39 (41.5%)	44 (46.8%)	5 (5.3%)	1 (1.1%)
Kittens					
Mean weight (SD) (kg)	–	–	1.04 (0.73)	0.65 (0.39)	0.95 (0)
Median weight (Q1, Q3) (kg)	–	–	0.96 (0.69, 1.28)	0.51 (0.31, 0.93)	0.95 (0.95, 0.95)
Number (%)	0 (0%)	0 (0%)	18 (54.6%)	14 (42.4%)	1 (3.0%)

Similar symbols (*, †, ‡) signifies significant differences in median body weights at *p* < 0.05.

**Table 2:** Association between animal weights and location of intravenous catheters utilized in puppies and kittens presented to a veterinary emergency room.

Location of catheter placement	Saphenous	Cephalic	Jugular
Puppies			
Mean weight (SD) (kg)	7.05 (4.10)	5.19 (3.40)	1.31 (1.14)
Median weight (Q1, Q3) (kg)	6.98 (3.68, 10.43) *	4.37 (2.65, 7.42) †	1.15 (0.54, 1.29) * †
Number (%)	4 (4.3%)	85 (90.4%)	5 (5.32%)
Kittens			
Mean weight (SD) (kg)	0.97 (0.33)	0.96 (0.64)	0.26 (0.04)
Median weight (Q1, Q3) (kg)	1.14 (0.59, 1.17)	0.93 (0.52, 1.26)	0.27 (0.23, 0.28)
Number (%)	3 (9.1%)	26 (78.8%)	4 (12.1%)

Similar symbols (*, †) signifies significant differences in median body weights at *p* < 0.05.

Only five catheters needed to be replaced during hospitalization due to the catheters not being patent. The median time for replacement was about 20 h. Eighty-one catheters were removed due to patient discharge, 7 catheters because of patient euthanasia and 1 after the patient suffered cardiopulmonary arrest while hospitalized.

### Feline data

**Figure 2:**
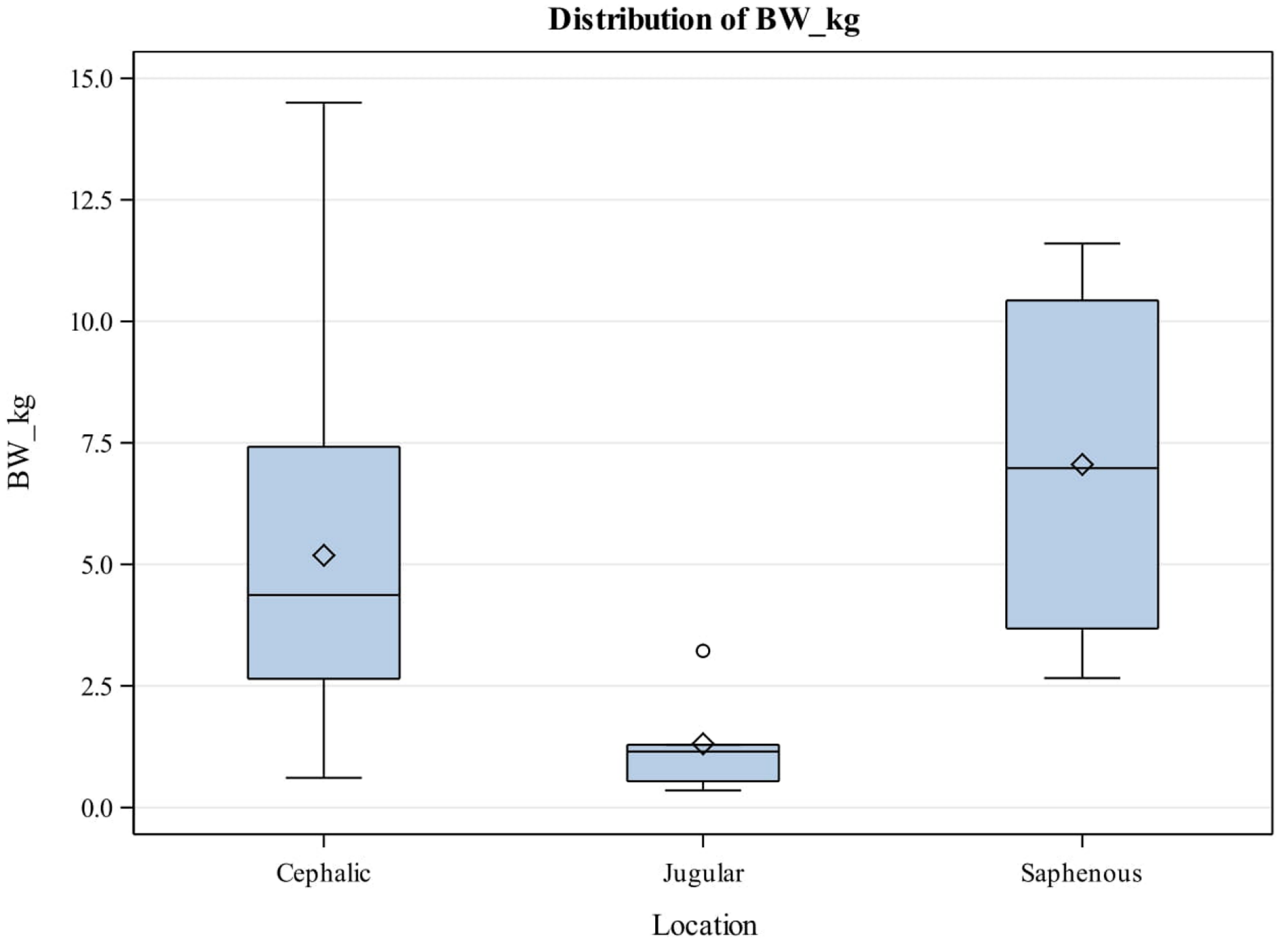
Box and Whisker plot showing relationship between body weight (kg) and catheter location in puppies. The center line represents the median value (Q2 or the 50th percentile) and the diamond represents the mean. The box indicates the lower (QI or the 25th percentile) and upper (Q3 or the 75th percentile) quartiles while the whiskers indicate the 5th and 95th percentiles. Values beyond the upper and lower boundaries are outliers.

Thirteen female, 15 male and 5 kittens whose sex were undetermined enrolled in the study. The most common breeds represented were Domestic Short Hair (26, 78.79%), Domestic Medium Hair (3, 9.09%), and Domestic Long Hair (2, 6.06%). Kittens had a median body weight of 8 weeks (Q1, Q3: 8, 10) with a median body weight of 0.92 kgs (0Q1, Q3: 0.43, 1.14). The most common reason for venous catheterization in kittens was for hospitalization (27, 81.82%), transfusions (4, 12.12%) and sedation (2, 6.06%). The most common catheter size utilized was 22G catheters (Table 1) with most catheters placed in the cephalic vein (26, 78.79%) (Table 2). No kitten had an 18G or 20G catheter placed. There was no significant association between the body weight and catheter size placed in kittens (Table 1). There was also no significant association between the kitten’s body weight and the location of the catheter placed (Table 2; Figure 2).

Only 1 catheter was reported to be replaced, about 15 h after the original catheter was placed. That catheter was removed for not being patent. Twenty catheters were removed due to patient discharge, 9 catheters were removed because of patient euthanasia and three after the patients suffered cardiopulmonary arrest while hospitalized.

## Discussion

The results of this retrospective study suggest that the cephalic vein is most utilized for intravenous catheter placement in pediatric puppies and kittens younger than 12 weeks of age. Anecdotally, the jugular vein appears to be preferred in pediatrics because of its larger size, which may allow easier access (18). However utilizing a short small bore catheter in the jugular vein has the limitations of being fraught to kinking due to the short length of the catheter and the animal’s neck position (19). In addition, placing a catheter in the external jugular vein may be more technically challenging, depending on individual venipuncture skills, and may delay life-saving intervention in that patient. Identification of extravasating infusions may also be difficult in catheters placed in jugular vein. While our hypothesis was that the jugular vein would be most used in pediatric patients, our results suggest that the cephalic vein was most utilized in our institution in 79 and 90% of pediatric kittens and puppies, respectively. This finding is especially profound in the kittens in this study with a median body weight of 0.92 kg, where the cephalic vein was still most used despite the small body weight. The results of this study suggest that the cephalic vein should be the initial choice for catheter placement in pediatric patients requiring venous access. However, the jugular vein may be considered initially in puppies and kittens with body weights around 1.2 kg and 0.3 kg respectively, although there was no significant association between venous access location and body weight in kittens. The lack of significant association between body weight and venous access location in kittens also suggests that a skilled operator can successfully place cephalic catheters in kittens regardless of the body weight, but due to the low sample size, this may represent a type 2 error.

In interpreting the results of this study, it is important to emphasize that we were not able to evaluate the number of attempts it took to place the catheters. It could not be determined if cephalic veins had been attempted in puppies and kittens that ultimately had venous access via the jugular vein or vice versa. Due to this limitation, a prospective study evaluating pediatric DiVA in animals would help clarify this.

In sick and critically ill neonatal patients, larger catheter sizes allow for rapid delivery of life-saving intervention, including intravenous fluids and blood products. Most puppies and kittens in this study had 22G catheters placed, which is capable of infusing approximately 36 mL/min in human patients (20), which is very sufficient for rapid infusions of fluids and blood products in pediatric veterinary patients. About 42% of kittens had 24G catheters placed, which is capable of delivering infusions at approximately 20 mL/min (21), which is also higher than the infusion delivery rates any of the pediatric patients represented in this study would require. Hence, the catheter size placed in the animals represented in this study were adequate for emergent infusions.

Limitations of this study include its retrospective nature, which did not allow for full evaluation of DiVA or rationale behind the choice of venous access placement or catheter size. It is also possible that because this is a single institutional study, the practice may have had a bias toward the use of cephalic veins for venous access. Finally, the technical skills and experience of the individuals who placed the catheter could not be determined as no identifying information was included in the medical records.

Overall, the cephalic vein is readily accessible for venous access in puppies and kittens under 12 weeks of age, across a wide range of body weights but the jugular vein may be most ideal for puppies and kittens under 1.2 kg and 0.3 kg, respectively. The most common catheter sizes utilized in this population of patients are 22G and 24G catheters, which are adequate sizes for resuscitation needs in pediatric canine and feline patients.

## Data Availability

The raw data supporting the conclusions of this article will be made available by the authors, without undue reservation.

## Footnotes

^1^Cornerstone Software. IDEXX Laboratories Inc., Westbook, Maine.

^2^Instinct Treatment Sheets. Instinct Science. Furlong, Pennsylvania.

^3^SAS 9.4: SAS Institute Inc., Cary, NC, United States.
